# Divergent Roles for TRAIL in Lung Diseases

**DOI:** 10.3389/fmed.2018.00212

**Published:** 2018-07-27

**Authors:** Adam T. Braithwaite, Helen M. Marriott, Allan Lawrie

**Affiliations:** Department of Infection, Immunity and Cardiovascular Disease, University of Sheffield, Medical School, Sheffield, United Kingdom

**Keywords:** TRAIL, TNF-related apoptosis-inducing ligand, pulmonary arterial hypertension, immune regulation, pulmonary vascular disease, pulmonary fibrosis, respiratory tract infections, chronic obstructive pulmonary diseases

## Abstract

The tumour necrosis factor-related apoptosis-inducing ligand (TRAIL) is a widely expressed cytokine that can bind five different receptors. TRAIL has been of particular interest for its proposed ability to selectively induce apoptosis in tumour cells. However, it has also been found to regulate a wide variety of non-canonical cellular effects including survival, migration and proliferation via kinase signalling pathways. Lung diseases represent a wide range of conditions affecting multiple tissues. TRAIL has been implicated in several biological processes underlying lung diseases, including angiogenesis, inflammation, and immune regulation. For example, TRAIL is detrimental in pulmonary arterial hypertension—it is upregulated in patient serum and lungs, and drives the underlying proliferative pulmonary vascular remodelling in rodent models. However, TRAIL protects against pulmonary fibrosis in mice models—by inducing apoptosis of neutrophils—and reduced serum TRAIL is found in patients. Conversely, in the airways TRAIL positively regulates inflammation and immune response. In COPD patients and asthmatic patients challenged with antigen, TRAIL and its death receptors are upregulated in serum and airways. Furthermore, TRAIL-deleted mouse models have reduced airway inflammation and remodelling. In the context of respiratory infections, TRAIL assists in immune response, e.g., via T-cell toxicity in influenza infection, and neutrophil killing in S. pneumoniae infection. In this mini-review, we examine the functions of TRAIL and highlight the diverse roles TRAIL has in diseases affecting the lung. Disentangling the facets of TRAIL signalling in lung diseases could help in understanding their pathogenic processes and targeting novel treatments.

## Introduction

**Figure 1:**
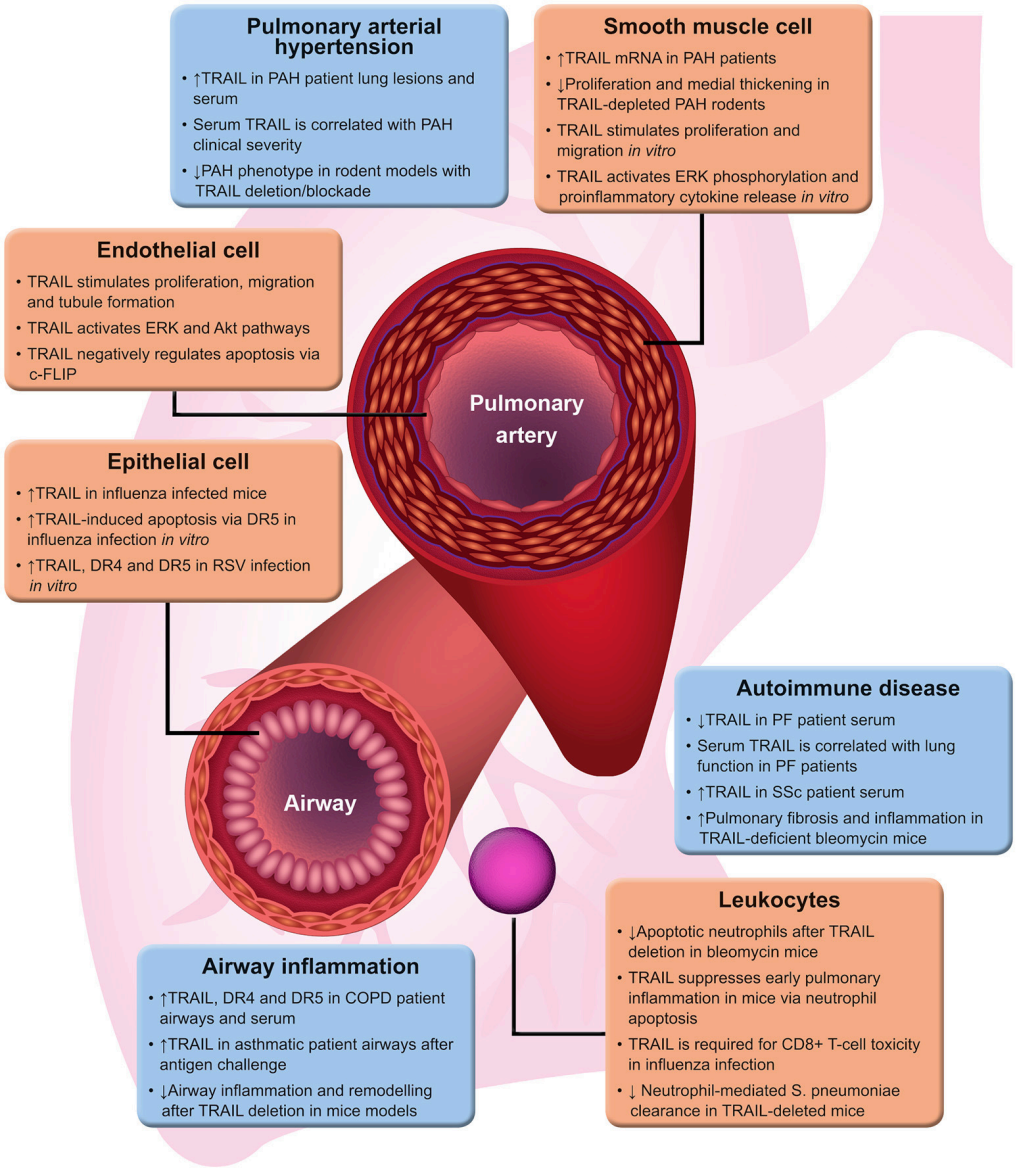
TRAIL functions in lung disease. A brief summary of evidence for the varied roles of TRAIL in different lung diseases. ↑ and ↓ represent up- and down-regulation, respectively. Akt, protein kinase B; COPD, chronic obstructive pulmonary disease; DR4/5, TRAIL death receptor 4/5; ERK, extracellular signal-regulated kinase; PAH, pulmonary arterial hypertension; PF, pulmonary fibrosis; RSV, respiratory syncytial virus; SSc, systemic sclerosis; TRAIL, tumour necrosis factor-related apoptosis-inducing ligand.

The tumour necrosis factor-related apoptosis-inducing ligand (TRAIL), also known as Apo2 ligand is an apoptosis-inducing cytokine that is expressed in most cell types. As its name suggests, TRAIL was primarily of particular interest for its ability to selectively induce apoptosis in tumour cells *in vitro* and *in vivo*, while apparently exhibiting minimal off-target effects (1–3). TRAIL-deficient mice are also more susceptible to tumour formation and metastasis (4), suggesting TRAIL has a protective role in cancer suppression. Consequently TRAIL signalling has been targeted for use in several anticancer therapies (5), however several types of cancer cells are resistant to TRAIL-induced apoptosis. In these cells, TRAIL can activate pro-inflammatory signalling pathways (6, 7), proliferation (8–10) and metastasis (11). The purpose of this mini-review is to discuss how the known function of TRAIL has evolved beyond apoptosis to these alternative effects and highlight the different roles TRAIL has in diseases affecting the lung (Figure 1), where TRAIL is widely expressed (12, 13). The better understanding of the diverse roles for TRAIL in lung disease could lead to the development of more effective, and novel treatments.

## TRAIL molecular signalling

TRAIL, a type II transmembrane protein, is a member of the death receptor ligand family; a subclass of the tumour necrosis factor family (14) and is widely expressed in a variety of human tissues, most predominantly in lung, spleen and prostate (14). TRAIL is proteolytically-cleaved and its extracellular domain can bind five TRAIL receptors: membrane-bound death receptors DR4 (TRAIL-R1) and DR5 (TRAIL-R2), membrane-bound decoy receptors DcR1 (TRAIL-R3), and DcR2 (TRAIL-R4) and the soluble decoy osteoprotegerin (OPG) (15–21) [TRAIL is conserved in mice—they have two decoy receptors and a single TRAIL death receptor, mDR5, which is more similar to DR5 than DR4 (22)].

**Figure 2:**
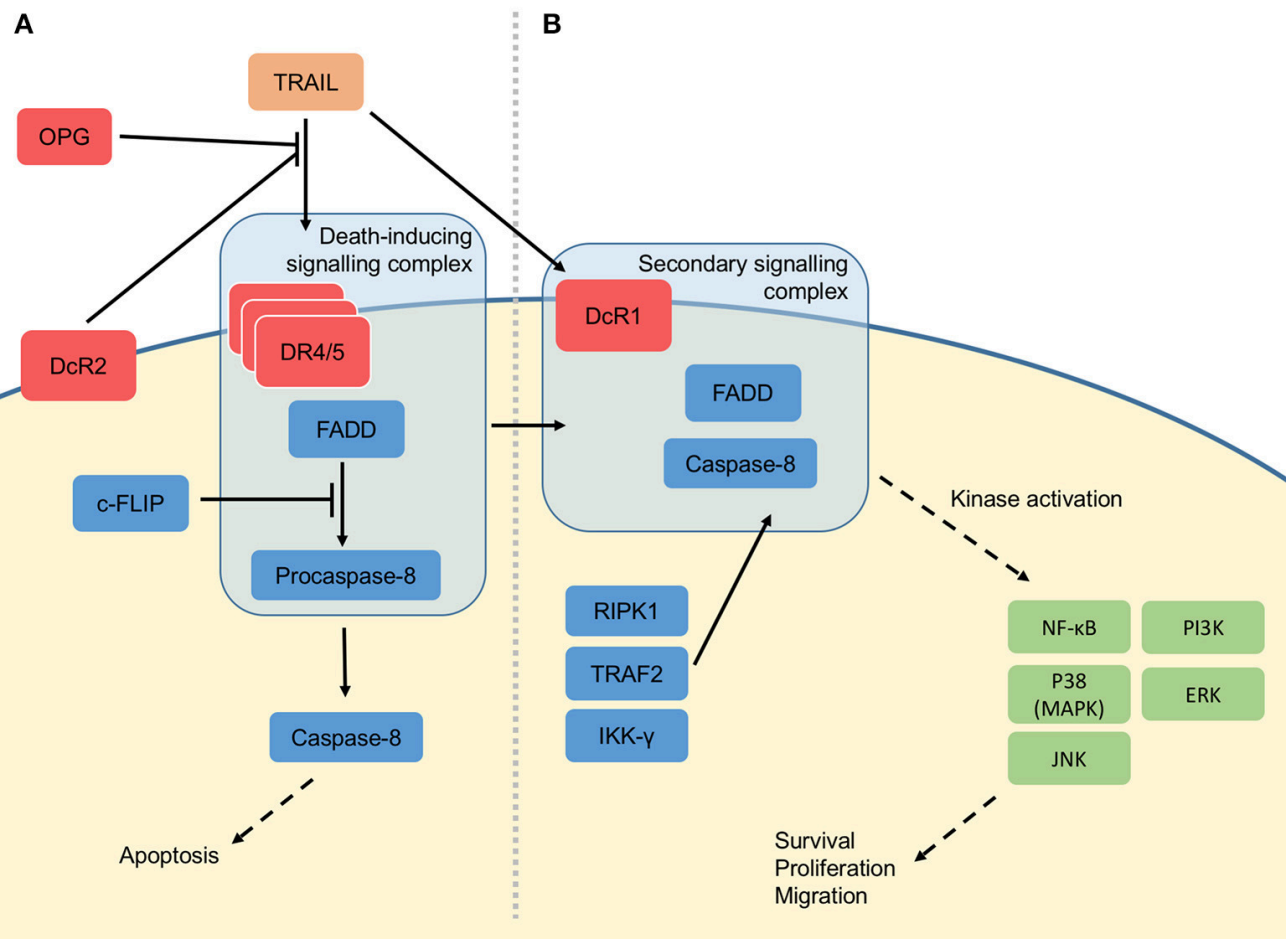
Molecular signalling of TRAIL. **(A)** Three proteolytically-cleaved tumour necrosis factor-related apoptosis-inducing ligand (TRAIL) proteins form a homotrimeric structure when binding death receptor 4 (DR4) or death receptor 5 (DR5) at the cell membrane. These are joined by Fas-associated death domain (FADD) and procaspase-8 to form the so-called death-inducing signalling complex (DISC). The DISC causes activation of the caspase cascade, leading to apoptosis of the cell. TRAIL may also bind the membrane decoy receptors (DcR1/2) or soluble osteoprotegerin (OPG), which do not contain a death domain, thus preventing TRAIL-induced apoptosis. Apoptosis can also be suppressed by (FADD-like interleukin-1β-converting enzyme)-inhibitory protein (c-FLIP), which inhibits the function of the DISC. **(B)** In the non-canonical signalling pathway, the receptor and ligand are thought to be lost, leaving FADD and caspase-8 to be joined by receptor-interacting serine/threonine-protein kinase 1 (RIPK1), TNF receptor-associated factor 2 (TRAF2) and inhibitor of NF-κB kinase subunit gamma (IKK-γ). This secondary signalling complex initiates protein kinase signalling pathways, leading to activation of kinases including nuclear factor kappa-light-chain-enhancer of activated B cells (NF-κB), p38 (mitogen-activated protein kinase; MAPK), c-Jun N-terminal kinase (JNK), phosphatidylinositide 3-kinases (PI3K) and extracellular signal-regulated kinase (ERK). The effects of these kinases include survival, proliferation and migration.

TRAIL is composed of 281 amino acids and forms a homotrimeric structure upon binding three receptor molecules (23). The death receptors DR4 and DR5 are type I transmembrane proteins containing a cytoplasmic death domain. In the canonical TRAIL apoptosis signalling pathway (Figure 2A), binding of death receptors by TRAIL leads to recruitment of Fas-associated protein with death domain (FADD), formation of a complex known as death-inducing signalling complex (DISC), activation of caspase-8 and subsequently downstream caspase-3 dependent apoptosis of the cell [Figure 1; (24–26)]. Unlike the TRAIL death receptors, the decoy receptor DcR1 has no death domain (15, 18) and DcR2 has a truncated, non-functional death domain (15, 17, 27). These decoy receptors, and additionally binding with lower affinity, the soluble OPG, are suggested to suppress apoptotic signalling by competitively binding TRAIL (28, 29).

Conversely, TRAIL can also stimulate pathways promoting cell survival, proliferation and migration via activation of kinase signalling pathways (Figure 2B) (30). This non-canonical signalling may depend on the formation of a secondary signalling complex after initial DISC assembly (31), recruiting other factors including FADD, Caspase 8, RIPK1, TNF receptor-associated factor 2 (TRAF2) and inhibitor of NF-κB kinase subunit gamma (IKK-γ). Activation of non-canonical TRAIL signalling pathways may also be regulated by expression of DcR1, as antibody neutralisation of this decoy receptor can inhibit TRAIL-induced cell proliferation (30). Downstream non-canonical signalling by TRAIL has been shown to be effected by activation of kinase signalling e.g., NF-κB, p38, c-Jun N-terminal kinase (JNK), phosphatidylinositide 3-kinases (PI3K), Akt, and extracellular signal-regulated kinases (ERK); leading to activation of gene transcription (32). By activating NF-κB, TRAIL can also modulate levels of FADD-like interleukin-1β-converting enzyme)-inhibitory protein [c-FLIP; (33)], a negative regulator of caspase-mediated apoptosis—a further mechanism by which a cell may deviate from pro-apoptotic to pro-survival signalling in response to TRAIL.

## Pulmonary arterial hypertension

TRAIL has been implicated in the pathobiology of pulmonary arterial hypertension (30, 34). This is indicated by elevated levels of soluble TRAIL found in the serum of PAH patients, and increased abundance of serum TRAIL, which is associated with worsened clinical severity (35). The pulmonary vasculature is complex, and many aberrant processes can lead to disease. PAH is a multifactorial disorder characterised by remodelling of the pulmonary arteries and a progressive increase in pulmonary vascular resistance, leading to raised afterload on the right ventricle and ultimately right heart failure (36). The most frequent alterations are sustained pulmonary vasoconstriction and remodelling of the pulmonary arteries and arterioles. The arterial remodelling is characterised by medial hypertrophy, intimal fibrosis and often the development of thrombotic or plexiform lesions (37). Together, these processes cause the occlusion of small pulmonary arteries. Combined with the muscularisation and progressive obliteration of distal vessels, the subsequent loss of cross-sectional area generates increased right ventricular afterload. At the cellular level, the neoplastic pathologies of PAH are thought to be driven by excessive proliferation of apoptosis-resistant endothelial cells (ECs), together with proliferation and migration of medial smooth muscle cells (SMCs) and fibroblasts.

TRAIL immunoreactivity has been shown in pulmonary vascular lesions from idiopathic PAH patients (13) and increased TRAIL mRNA expression is detected in the lungs of rodent models of PAH (35, 38). Furthermore, TRAIL has been demonstrated—by knockout and by inactivation—as necessary for the development of PAH in multiple pre-clinical models of PAH (30). Reversal of established PAH in rodent models was also demonstrated by administration of an anti-TRAIL antibody (30). TRAIL knockout also had a similar protective effect in a Sugen5416 and hypoxia mouse model of PAH (34). Increased TRAIL, DR4 and DcR1 mRNA levels have been detected in explanted pulmonary artery SMCs from idiopathic PAH patients, compared to healthy control cells (30). Additionally, TRAIL depletion or blockade in rodent models of PAH is associated with reduced pulmonary arterial remodelling with fewer proliferating pulmonary artery SMCs (30, 34). This evidence indicates that TRAIL is a key promoter of the pulmonary arterial SMC proliferation associated with the pathogenic vascular remodelling in PAH. Recombinant TRAIL was also shown to induce proliferation and migration of idiopathic PAH patient pulmonary artery SMCs *in vitro*, via phosphorylation of ERK1/2 (30). The pro-proliferative effect of TRAIL was reversed by the addition of DcR1 neutralising antibody, suggesting this decoy receptor is essential to non-canonical TRAIL signalling in pulmonary artery SMCs. Other studies have similarly demonstrated that TRAIL can stimulate proliferation and migration of vascular SMCs via non-canonical kinase signalling cascades (39, 40). Additionally, following activation of NF-κB, TRAIL has been shown to stimulate production and release of pro-inflammatory cytokines in vascular SMCs (41).

EC dysfunction is another key aspect of the angioproliferative state of pulmonary arteries in PAH. Several studies have demonstrated that TRAIL can stimulate angiogenic processes in vascular ECs *in vitro*, including proliferation (33, 42, 43), migration (33, 43, 44) and tubule formation (43). Similarly to non-canonical TRAIL signalling in SMCs, its angioproliferative effect in ECs has been linked to activation of Akt and ERK pathways (42), as well as upregulation of DcR2 (45). Conversely, TRAIL has also been demonstrated to have apoptotic (12, 46) and anti-angiogenic (47) effects on vascular ECs. The reason for this disparity is unclear, although each of these studies used a relatively high concentration of recombinant TRAIL (100 ng/ml), suggesting the pro-angiogenic signalling in endothelium may preferentially occur at lower TRAIL concentrations. In Cantarella et al. (33), high levels of TRAIL were shown to induce caspase 8-mediated apoptosis of ECs, whereas low levels of TRAIL were pro-angiogenic. Interestingly, these dose-dependent opposing effects of TRAIL in ECs were linked to modulation of levels of c-FLIP, a procaspase-8 homolog and negative regulator of apoptosis (33).

## Autoimmune disease

TRAIL is now known to have crucial functions in regulation of inflammation and immune response. These systems are significant in the pathogenesis of many forms of lung disease, including autoimmune disorders and respiratory infection in addition to pulmonary vascular disease (Figure 2). A role for TRAIL in regulating inflammation via apoptosis was highlighted in a knockout of the mouse TRAIL death receptor, as in addition to tumour formation, the mice were prone to chronic inflammation (48). Additionally, TRAIL has been demonstrated to suppress the early inflammatory response via apoptosis of neutrophils (49).

PAH is an associated complication in autoimmune disease, e.g., 7–12% of patients with systemic sclerosis (SSc) develop PAH (50, 51). SSc is a heterogeneous autoimmune disorder, characterised by tissue fibrosis and vascular injury. Pulmonary fibrosis (PF) is a condition often found in interstitial lung disease and autoimmune disorders of the connective tissue, including SSc and rheumatoid arthritis. Elevated serum TRAIL levels have been found in SSc patients compared to healthy controls, in addition to being elevated in SSc patients with either PAH or PF compared to those without pulmonary involvement (52), suggesting that TRAIL may also may play a key role. In contrast, soluble TRAIL has been found at lower levels in the serum of patients with the idiopathic form of PF than health controls (53). Within the idiopathic PF patient group, lung function—shown by transfer factor of the lung for carbon monoxide—was correlated with serum levels of TRAIL, suggesting it may have a protective role in idiopathic PF (53). Furthermore, a direct link to PF pathobiology is illustrated in TRAIL-deficient mice, where fibrosis in the bleomycin model of PF was enhanced in comparison to wild-type mice (53). In this model, TRAIL deletion also increased pulmonary inflammation (neutrophil counts in bronchoalveolar lavage fluid). The inflammatory phenotype in TRAIL knockout mice was accompanied by a reduced number of apoptotic cells in lung tissue, with a corresponding reduction of apoptotic neutrophils in bronchoalveolar lavage fluid. This suggests that TRAIL-mediated apoptosis of neutrophils is a protective process in this form of PF.

## Airway inflammation

Contrary to its protective effect in idiopathic PF, TRAIL appears to have a detrimental role in the context of both acute and chronic airway inflammation, by upregulating inflammation and autoimmune responses (Figure 2). TRAIL is elevated in bronchoalveolar lavage fluid from asthmatic patients following antigen challenge, and isolated eosinophils express more TRAIL and DcR2, but less DR4 and DR5 (54). Deletion of the TRAIL gene in mice diminishes airway hyper-reactivity, inflammation and remodelling in an ovalbumin-induced model of allergic asthma (55, 56) and a rhinovirus-induced asthma model (57). Additionally, chronic asthmatic inflammation, remodelling and lung function are worsened by TRAIL deletion in mice infected as neonates with chlamydia (58).

Prolonged exposure to irritants and inflammation can lead to chronic obstructive pulmonary disease (COPD). A role for TRAIL in COPD has been highlighted by its elevated levels in the lungs of COPD patients. One study found increased TRAIL, DR4, DR5, and DcR1 protein in lung parenchyma from COPD patients (59). Higher levels of TRAIL, DR4, and DR5 mRNA were also found in airway epithelial brushing of COPD patients compared to healthy controls (60). Another study found increased levels of serum TRAIL and DR5 in COPD patients compared to healthy controls (61). Additionally, with the COPD patient group serum levels of TRAIL and DR5 were found to be inversely correlated with forced expiratory volume (61). Inflammation and alveolar cell apoptosis are key processes in many forms of COPD. A pro-apoptotic function of TRAIL in COPD was originally suggested, as emphysematous lung tissue is more sensitive to TRAIL-induced apoptosis than health lung (62). However, a pro-inflammatory element may also be important. In a chronic cigarette smoke-exposure mouse model of COPD, TRAIL mRNA, and protein expression was increased in the airway epithelium and parenchyma, and in mice with TRAIL deletion, airway inflammation—as well as remodelling—was reduced (60). The activation by TRAIL of both apoptotic and inflammatory pathways within COPD highlights its varied roles and how specific cell types are targeted—whether or not this is this mediated by differential receptor expression or some other mechanism remains unclear.

## Respiratory infection

In lower respiratory tract infections, TRAIL has differing roles in immune response and damage to host tissues (Figure 2). Apoptosis of virus-infected cells is a key mechanism for clearance of viral infection and *in vitro*. In the context of influenza infection, TRAIL-induced apoptosis of human lung alveolar epithelial cells is enhanced; an effect which is inhibited by blocking DR5 (63). Similarly, TRAIL, DR4, and DR5 are strongly upregulated in response to respiratory syncytial virus infection in pulmonary epithelial cells, leading to increased sensitivity to apoptosis (64). In animal models, TRAIL expressed by CD8+ T-cells has been demonstrated as essential for viral immunity, with TRAIL knockout mice exhibiting increased influenza-associated morbidity and reduced CD8+ T-cell cytotoxicity (65–67). DR5 expression was also shown to be upregulated in influenza-infected pulmonary epithelial cells *in vivo* (63, 65).

In opposition to its protective role in viral clearance, other studies have shown that TRAIL expressed by macrophages is instrumental in damage to airways caused by apoptosis of alveolar epithelial cells in influenza infection (68, 69). Deletion of TRAIL in mice led to a reduction in mortality and the alveolar epithelial apoptosis and alveolar leakage associated with influenza virus pneumonia (68). This highlights an interesting situation whereby TRAIL death signalling may be used for host for viral clearance, while also assisting in viral infection via tissue damage. TRAIL has also been demonstrated as important in immune response to bacterial respiratory infection. In the context of Streptococcus pneumoniae infection, deletion of TRAIL in mice reduces bacterial clearance in the lungs and worsens survival—an effect that is reversed by treatment with TRAIL or DR5 agonist antibody (70). In the same study, neutrophils were found to be the key source of TRAIL (70).

## Conclusions

As highlighted in this mini-review, TRAIL is multifaceted in a variety of lung diseases. TRAIL also has the ability to function as either pro-apoptotic or pro-survival depending on the cells type, and receptor expression on local tissue to mediate either protective or pathogenic mechanisms. The exact mechanism by which TRAIL modulates these functions is not fully understood, although regulation of TRAIL, and its cleavage, as well as the expression of receptors by specific cell types is clearly important in determining its effects. Further work is required to fully elucidate the divergent roles of TRAIL to gain a better understanding of the role it plays in underlying processes of lung disease, and its potential as a therapeutic agent—or target—depending on disease context.

## Author contributions

AB was involved in conception and design of the work, drafting the article and final approval of the version to be published. HM and AL were involved in critical revision of the article and final approval of the version to be published.

### Conflict of interest statement

The authors declare that the research was conducted in the absence of any commercial or financial relationships that could be construed as a potential conflict of interest.

## Abbreviations

COPD: Chronic obstructive pulmonary disease
DcR1/2: Decoy receptor 1/2
DISC: Death-inducing signalling complex
DR4/5: Death receptor 4/5
EC: Endothelial cell
PAH: Pulmonary arterial hypertension
PF: Pulmonary fibrosis
SMC: Smooth muscle cell
SSc: Systemic sclerosis.
